# Adaptation of motor unit contractile properties in rat medial gastrocnemius to treadmill endurance training: Relationship to muscle mitochondrial biogenesis

**DOI:** 10.1371/journal.pone.0195704

**Published:** 2018-04-19

**Authors:** Katarzyna Kryściak, Joanna Majerczak, Jakub Kryściak, Dawid Łochyński, Dominik Kaczmarek, Hanna Drzymała-Celichowska, Piotr Krutki, Anna Gawedzka, Magdalena Guzik, Michał Korostynski, Zbigniew Szkutnik, Elżbieta Pyza, Wiesława Jarmuszkiewicz, Jerzy A. Zoladz, Jan Celichowski

**Affiliations:** 1 Department of Neurobiology, Poznan University of Physical Education, Poznan, Poland; 2 Department of Muscle Physiology, Faculty of Rehabilitation, University School of Physical Education, Krakow, Poland; 3 Department of Physiology, Poznan University of Physical Education, Poznan, Poland; 4 Department of Musculoskeletal Rehabilitation, Poznan University of Physical Education, Poznan, Poland; 5 Department of Biochemistry, Poznan University of Physical Education, Poznan, Poland; 6 Department of Molecular Neuropharmacology, Institute of Pharmacology, Polish Academy of Sciences, Krakow, Poland; 7 Faculty of Applied Mathematics, AGH-University of Science and Technology, Krakow, Poland; 8 Department of Cell Biology and Imaging, Institute of Zoology, Jagiellonian University, Krakow, Poland; 9 Department of Bioenergetics, Adam Mickiewicz University, Poznan, Poland; Universidad Pablo de Olavide, SPAIN

## Abstract

This study aimed at investigating the effects of 2, 4 and 8 weeks of endurance training on the contractile properties of slow (S), fast fatigue resistant (FR) and fast fatigable (FF) motor units (MUs) in rat medial gastrocnemius (MG) in relation to the changes in muscle mitochondrial biogenesis. The properties of functionally isolated MUs were examined *in vivo*. Mitochondrial biogenesis was judged based on the changes in mitochondrial DNA copy number (mtDNA), the content of the electron transport chain (ETC) proteins and PGC-1α in the MG. Moreover, the markers of mitochondria remodeling mitofusins (*Mfn1*, *Mfn2*) and dynamin-like protein (*Opa1*) were studied using qPCR. A proportion of FR MUs increased from 37.9% to 50.8% and a proportion of FF units decreased from 44.7% to 26.6% after 8 weeks of training. The increased fatigue resistance, shortened twitch duration, and increased ability to potentiate force were found as early as after 2 weeks of endurance training, predominantly in FR MUs. Moreover, just after 2 weeks of the training an enhancement of the mitochondrial network remodeling was present as judged by an increase in expression of *Mfn1*, *Opa1* and an increase in PGC-1α in the slow part of MG. Interestingly, no signs of intensification of mitochondrial biogenesis assessed by ETC proteins content and mtDNA in slow and fast parts of gastrocnemius were found at this stage of the training. Nevertheless, after 8 weeks of training an increase in the ETC protein content was observed, but mainly in the slow part of gastrocnemius. Concluding, the functional changes in MUs’ contractile properties leading to the enhancement of muscle performance accompanied by an activation of signalling that controls the muscle mitochondrial network reorganisation and mitochondrial biogenesis belong to an early muscle adaptive responses that precede an increase in mitochondrial ETC protein content.

## Introduction

Endurance training enhances muscle performance and resistance to fatigue [1–5]. Prolonged periods of endurance training have been reported to modify the activity and content of proteins associated with ATP usage, such as sarco/endoplasmic reticulum (SR) Ca^2+^-ATPase (SERCA) pumps [6–8], parvalbumin and myosin heavy chain (MyHC) isoforms [9,10] as well as proteins involved in the ATP production system, including electron transport chain proteins in the mitochondria [3,11,12]. It has been shown however, that during endurance training the process of fast-to-slow transition in skeletal muscle does not occur simultaneously, but in an ordered sequence and the changes in the expression of the proteins involved in the Ca^2+^ handling (including Ca^2+^ pumps in SR and parvalbumin—the Ca^2+^ binding protein in fast skeletal muscle) precede the changes in the myofibryllar proteins [9,13]. It has been reported that the transition of myosin heavy chain isoforms and fiber types in most cases is limited to the fast fiber subtypes [14] and might be detected not earlier than after several weeks of training [9,10]. The most recent studies have shown that endurance training also increases mitochondrial efficiency in the trained muscles, i.e., enhances the mitochondrial phosphate/oxygen ratio (P/O) [15]. It is thought that training-induced increase in muscle mitochondrial biogenesis is the key adaptive muscle response to endurance training, leading to improved muscle metabolic status and enhanced muscle endurance [1,16]. The effect of endurance training on *de novo* mitochondrial biogenesis is still poorly documented. Some recent studies put some new light on this issue. For example, Meinild Lundby et al. [17] have shown that six weeks of endurance training increases mitochondrial volume density (by about 40%) in humans, however, this training-induced augmentation in mitochondrial volume is a result of an enlargement of existing mitochondria and is not related to an increase in mitochondria content (*de novo* biogenesis).

Nevertheless, it is well documented that endurance training increases muscle mitochondrial oxidative capacity as judged by an increase in mitochondrial enzymes activity/content such as citrate synthase and cytochrome-c oxidase [12,18]. This muscle adaptive response leads to an enhancement of muscle metabolic stability i.e., an attenuated decrease in ΔG_ATP_ and a lesser increase in muscles metabolites such as PCr, Pi, ADP and H^+^ during exercise at a given power output [8,12,19]. It should be underlined, that at early stages of the training, enhanced muscle metabolic stability during exercise may precede an increase in the activity of specific enzymes, including those involved in glycolysis, β-oxidation and the citric acid cycle [8,12,20,21].

Surprisingly, however, little is known about the effect of endurance training on the characteristics of motor units (MUs) in different muscles (slow and fast). MUs are the smallest functional elements of neuromuscular system, each MU is formed by one motor neuron and a group of muscle fibres innervated exclusively by that neuron. Many studies have demonstrated that MUs have ability to adapt to altered muscle use and/or level of muscle activity, defined as MU plasticity [22–25]. In research conducted on animals, it has been demonstrated that the three main physiological types of MUs (fast fatigable, FF; fast fatigue resistant, FR; and slow, S) react differently to changes in muscle activity, i.e. contractile properties of FF, FR and S types of MUs located in the same muscle may change with varying degrees, frequently even in opposite directions in response of the same change in muscle activity (for details see [26–31]). Therefore, knowledge of changes in the functional properties of MUs is crucial for understanding the adaptations of skeletal muscle, for example to endurance training. Some authors of human experiments have suggested a change in the recruitment and discharge rates of MUs in athletes due to endurance training in both electromyographic studies [2,32] and reflex studies [33–35], what was supported by studies of electrophysiological properties of motoneurons in animal experiments [36,37]. However, there have been no reports focused on endurance training-induced changes in contractile properties of MUs, in particular with respect to their physiological types.

In the present study, we aimed to answer the question whether endurance training-induced functional changes in MUs are associated with training-induced enhancement in mitochondrial biogenesis. Based on our previous reports [8,12], we hypothesized that the characteristic functional changes in MUs precede changes in mitochondrial biogenesis. Using a rodent model, we electrophysiologically investigated time-dependent changes in the contractile properties of the three types of MUs in the hind limb muscle (medial gastrocnemius, MG) induced by endurance training. Furthermore, we evaluated the effect of 2, 4 and 8 weeks of the endurance training on markers of mitochondrial remodelling (*Mfn1*, *Mfn2*, *Opa1*) and mitochondrial biogenesis (mtDNA/nDNA, ETC proteins content, PGC-1α) in MG.

## Materials and methods

Experiments were performed on 56 adult male Wistar rats. Animals were randomly assigned to four experimental groups, consisting of an untrained, sedentary—control (C) group (n = 14, six of which were subjected to electrophysiological experiments; mean age 163 ± 24 days) and three groups of rats trained on a treadmill: 2 weeks (2W; n = 14, six of which were subjected to electrophysiological experiments; mean age 173 ± 16 days), 4 weeks (4W; n = 14, six of which were subjected to electrophysiological experiments; mean age 156 ± 12 days), and 8 weeks (8W; n = 14, five of which were subjected to electrophysiological experiments; mean age 188 ± 11 days). During the experiments, all groups of animals were treated equally and housed under the same conditions. Rats were kept in standard laboratory cages (two per cage) in an animal house with a 12:12 light/dark cycle, controlled temperature (22 ± 2°C) and humidity (55 ± 10%). All rats had unrestricted access to standard laboratory food and tap water throughout the study period, ensuring a balanced nutrient diet.

All experimental procedures and animal care were performed in accordance with the Guiding Principles for the Care and Use of Animals in the Field of Physiological Sciences, European Union guidelines, and the Polish Law on the Protection of Animals. All experimental procedures were approved by the Local Ethics Committee on Animal Research in Poznan (Permission Number: 3/2013). All efforts were made to minimize suffering of animals.

### Treadmill training

Rats were trained on an electrical treadmill for small rodents (Exer-6M Treadmill; Columbus Instruments, Ohio, USA). Mild electric shocks were used to motivate rats and to ensure a continuous run. Training of the rats was conducted 5 days per week, at the same time each day. Due to the nocturnal activity of the animals, a reversed day/night cycle was introduced in the animal room, and training took place under weak red light. For all experimental groups, the body mass of animals was measured every week before training.

The intensity of training was lowest during the first week in order to habituate rats to the treadmill, consisting of: (1) 5–10 min slow running at 5–10 m min^-1^ with intervals of 5–10 s of progressively increased treadmill speed, up to 30–40 m min^-1^, followed by a decrease in velocity, which was repeated 10–20 times; (2) 60 min rest; (3) repeat of the first step of the procedure.

In subsequent weeks, training was conducted according to the following protocol: second week, 40 min continuous run at 30 m min^-1^ velocity; third week, 60 min continuous run at 30 m min^-1^; fourth week, 60 min continuous run at 30 m min^-1^, with acceleration every 10 min to 35 m min^-1^ for 30-s intervals (six accelerations at 10, 20, 30, 40, 50 and 60 min); fifth and sixth week, 60 min continuous run at 30 m min^-1^, with acceleration every 10 min to 40 m min^-1^ for 30-s intervals (six accelerations at 10, 20, 30, 40, 50 and 60 min); seventh and eighth week, 80 min continuous run at 30 m min^-1^, with acceleration every 13 min to 40 m min^-1^ for 30-s intervals (six accelerations at 13, 26, 39, 52, 65 and 78 min) (S1 Table). This training schedule was adapted from Dudley et al. [38] and was similar to the training protocol previously used by our research group [15] shown to obtain a significant acceleration in the biogenesis of mitochondrial proteins in the limb muscles of trained rats.

### Surgical preparation

All rats were anesthetized with pentobarbital sodium (initial i.p. dose of 60 mg kg^-1^, supplemented with additional doses of 10 mg kg^-1^ h^-1^ after 2 hours). The depth of anaesthesia was controlled by monitoring the pinna and withdrawal reflexes. The MG muscle from the right leg was dissected for muscle protein studies. The red (slow, MGS) and white (fast, MGF) portions of the MG muscle were carefully separated and immediately frozen. The MGF is a part located distally, predominantly corresponding to the distal compartment of the muscle [39] which consists of fast-twitch glycolytic fibres (20% type IIX and 80% IIB muscle fibres). The MGS is a deep and proximal part, corresponding to the proximal compartment. In rat this compartment contains about 20% oxidative fibres (10% type I muscle fibres, 10% type IIA, 45% type IIX and 35% type IIB) [40–42].

For the electrophysiological studies on MUs (n = 23), the spinal cord was exposed by a laminectomy over L2-S1 segments, the dura mater was removed, and the ventral roots of the spinal nerves were cut close to the spinal cord. The L5 or L4 ventral roots were carefully split into very thin filaments (single motor axons were isolated), which were subsequently stimulated electrically (rectangular pulses of 0.1 ms duration with varying amplitude of up to 0.5 V). The MG muscle of the left hind limb was partly isolated from the surrounding tissues (supplying blood vessels, proximal attachment and nerve branches of the MG muscle were left intact). The remaining muscles of the left hind limb that were innervated by the sciatic nerve were denervated.

The animal and operated hind limb were immobilized with steel clamps attached to the tibia, the sacral bone and the L1 vertebra. The Achilles tendon was connected to the inductive force transducer, and the MG muscle was stretched with a passive tension of up to 100 mN, previously reported as optimal for generating the highest isometric twitch force [43]. The MU action potentials were recorded using silver-wire bipolar electrodes that had been inserted into the MG muscle. The muscle, the exposed part of the spinal cord and the ventral roots were immersed in paraffin oil. Its temperature was maintained at 37 ± 1°C using an automatic heating system.

### Motor units stimulation, recording and analysis

The single MU activity was achieved when at increasing stimulation evoked twitches and muscle fibre action potentials were of the “all or none” type. The force and MU action potentials were observed on the oscilloscope and stored on a computer disc using an analog-to-digital converter (RTI-800 Utilities, Analog Devices, Norwood, MA, USA). Sampling rates of 1 kHz for force and 10 kHz for action potentials were used.

The same stimulation protocol was applied for each isolated MU to measure: (1) the averaged twitch contraction (five pulses at 1 Hz); (2) the fused tetanus (200-ms train of stimuli at 150 Hz); (3) series of tetani at progressively increasing stimulation frequencies (500-ms trains of stimuli at 5, 10, 15, 20, 30, 40, 50, 60, 75, 100 and 150 Hz at 10-s intervals); (4) the averaged twitch contraction (five pulses at 1 Hz); and (5) the fatigue test (trains of 14 pulses at 40 Hz, repeated every 1 s for 3 min) [44]. There were 10-s intervals between each of the five steps of the protocol described above. At the end of the experiment, animal was sacrificed by a lethal dose of pentobarbital sodium (180 mg kg^-1^).

The following contractile parameters of MUs were measured: (1) the twitch contraction time (CT; measured from the beginning of the contraction to the force peak); (2) the twitch half-relaxation time (HRT; measured from the force peak to half of this value); (3) the twitch force (TwF; measured from the baseline to peak force); (4) the maximal tetanic force (TetF; measured from the baseline to the peak of fused tetanic contraction at 150 Hz); (5) the twitch-to-tetanus ratio (Tw/Tet; ratio of the twitch force to the maximal tetanic force); (6) the post-tetanic potentiation (PTP; measured as the ratio of the twitch force, recorded in the fourth step of the stimulation protocol, to the initial twitch force, measured in the first step of the stimulation protocol). For each MU, the fatigue index (FatI) was calculated as a ratio of the force measured 2 min after the maximal initial force to the maximal force recorded at the beginning of the fatigue test (the fifth step of the stimulation protocol) [45,46]. Additionally, for each MU during the fatigue test forces developed in each fifth contraction within first 30 seconds and later within each tenth contraction were measured.

Classification of MUs as either slow or fast was based on the 20 Hz tetanus index, calculated as a ratio of the force of the last contraction within the tetanus at 20 Hz to the force of the first contraction within the tetanus [47]. Using the 20 Hz index, ratios of less than or equal to 2.0 represent fast MUs, and those higher than 2.0 represent S MUs (S1 File). Additionally, the fatigue index was used for further division of fast MUs into FF and FR. Fast MUs with the index below 0.5 were classified as FF, whereas those with the index above 0.5 were considered to be FR type (S1 File) [46,48,49].

The force-time area (FTA) corresponding to one stimulus (measured from the minimum force prior to the second to last stimulus to the minimum force prior to the last stimulus) [50,51] was analysed for all MUs in contractions induced at 1, 5, 10, 15, 20, 30, 40, 50, 60, 75, 100 and 150 Hz. The contraction with the maximum FTA was considered “the optimal tetanus”, and the relative force was calculated for these contractions.

The force-frequency curves were plotted for all MUs (using forces recorded during the third step of the stimulation protocol). For these curves, two parameters were calculated: the slope of a steep part of the curve, at 60% of the maximum force (representing the ability of the MU to increase the force in response to an increase in the stimulation frequency by 1 Hz), and the 60% Fmax, that is the stimulation frequency corresponding to 60% of the maximum force [46,52,53].

### RNA isolation and quantitative PCR

Before protein and RNA analyses, muscle samples i.e. fast (MGF) and slow (MGS) parts of gastrocnemius, were divided into two portions (one for RNA and one for protein analysis) using nitrogen-cooled instruments, which were stored at -80°C until further analysis. Analysis of *Myh7* (myosin heavy chain type 1), *Atp2a2* (sarco/endoplasmic reticulum Ca^2+^-ATPase, SERCA2), *Pvalb* (parvalbumin), *Opa1* (mitochondrial dynamin-like GTPase 1), *Mfn1* (mitofusin 1) and *Mfn2* (mitofusin 2) gene expression was performed in the MGF and in the MGS. The mRNA abundance levels of the selected transcripts were measured in the muscle samples. Frozen tissue samples were homogenized in 1 ml Trizol reagent (Invitrogen, Carlsbad, CA, USA), and total RNA was extracted according to the manufacturer’s protocol. The RNA concentration was measured using a ND-1000 Spectrometer (NanoDrop Technologies Inc., Montchanin, DE, USA). The quality of RNA was determined using an RNA 6000 Nano LabChip Kit (Agilent, Palo Alto, CA, USA) and measured using an Agilent Bioanalyzer 2100 (Agilent, Palo Alto, CA, USA). Reverse transcription was performed with Omniscript reverse transcriptase enzyme (Qiagen Inc., Valencia, CA, USA) at 37°C for 60 minutes. The qPCR reactions were performed using Assay-On-Demand TaqMan probes (*Myh7*, Rn01488777_g1; *Atp2a2*, Rn00568762_m1; *Pvalb*, Rn00574541_m1; *Opa1*, Rn00592200_m1; *Mfn1*, Rn00594496_m1 and *Mfn2*, Rn00500120_m1) according to the manufacturer’s protocol, (Thermo Fisher Scientific, Paisley, UK) and were run on a CFX96 Real-Time system (Bio-Rad, Foster City, CA, USA). Approximately 50 ng of cDNA synthesized from a total RNA template (isolated from an individual animal) was used for each reaction. Expression of the reference gene hypoxanthine phosphoribosyltransferase 1 (*Hprt1*, Rn01527840_m1) was quantified to control for variation in cDNA amounts. The abundance of RNA was calculated as 2^-(threshold cycle)^.

### Mitochondrial DNA (mtDNA) content

DNA was isolated from the MG (MGF and MGS, separately) using QIAamp DNA Mini Kit (Qiagen Inc., Valencia, CA, USA) according to the manufacturer’s protocol, and quantified by spectrophotometry using a NanoDrop ND-1000 Spectrometer (NanoDrop Technologies, Inc.). The mtDNA content was measured using qPCR with a CFX96 Real-Time system (Bio-Rad) [54]. TaqMan assay (Thermo Fisher Scientific) targeting *Atp6* mitochondrial gene was used to quantify the mtDNA. The primers and probe for qPCR analysis of mtDNA were designed using File Builder 3.1, and ordered as a custom TaqMan assay (Thermo Fisher Scientific). The TaqMan probe (assay ID CCX00SS, MT-ATP6) was labeled with the FAM fluorescent reporter. The quantity of mtDNA was corrected by simultaneous measurement of the single-copy nuclear *Tert* gene. A custom TaqMan assay was used to quantify the nuclear DNA (nDNA) (Custom TaqMan^®^ Copy Number Assay, rat_Tert_CCLJI3S; Thermo Fisher Scientific). The 20-μl PCR reaction contained 10 μl TaqMan Universal PCR Master Mix (Thermo Fisher Scientific), 1 μl of TaqMan primers and probe (20X) mix, and 5–10 ng of total genomic DNA. The PCR conditions were 2 min at 50°C then 10 min at 95°C, followed by 40 cycles consisting of 15 s of denaturation at 95°C and 60 s of annealing/extension at 60°C. Calibration curves were used to quantify mtDNA and nDNA copy numbers, which were based on the linear relationship between the crossing point cycle values and the logarithm of the starting copy number.

### Protein extraction and Western immunoblotting analysis

Muscle samples from the MGS and MGF parts of the MG, collected in the first step of the extraction procedure, were ground into a fine powder using a nitrogen-cooled mortar and pestle. The muscle samples were then ultrasonicated (UP 50H sonicator; Hielscher Ultrasonics GmbH, Teltow, Germany) on ice with a buffer consisting of 62.5 mM Tris (pH 6.8), 10% glycerol, 2.5% SDS and a protease inhibitor cocktail (Protease Inhibitor Cocktail 78415; Thermo Scientific, Wilmington, DE, USA). The sonicated samples were then incubated for 1 h under gentle agitation (HulaMixer, Invitrogen) at room temperature. Samples were then centrifuged for 30 min at 25000 × *g*, and the supernatant was transferred into fresh microcentrifuge tubes. The protein concentration in the sample extracts was measured using a NanoDrop 2000 UV-Vis Spectrophotometer (Thermo Scientific). The extracts were stored at -80°C until further analysis.

For detection of the content of subunits of mitochondrial complexes equal amounts of muscle samples (30 μg total protein), with addition of 2.5% 2-mercaptoethanol were loaded into gradient 4–20% Mini-PROTEAN^®^ TGX^™^ precast gels (BioRad, Hercules, CA, USA). Additional gels were loaded with the same amount of total protein (30 μg) from the same samples to measure β-actin as a loading control. To eliminate differences between gels resulting from the transfer, the internal standard (rat gastrocnemius muscle sample, 30 μg total protein) was applied on each gel.

For the detection of PGC-1α and GAPDH in the slow part of medial gastrocnemius (MGS) 20 μg total protein with addition of 2.5% 2-mercaptoethanol were loaded into gradient 4–20% Mini-PROTEAN^®^ TGX^™^ precast gels (BioRad, Hercules, CA, USA) after boiling the samples (5 min at 95°C).

**Fig 1:**
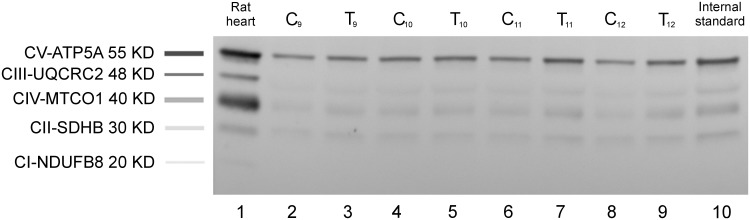
Respiratory chain proteins subunits in MGF of control and trained animals. Western blot analysis of protein extract from the fast part of the medial gastrocnemius (MGF) of control (C_9_ –C_12_) and trained rats (T_9_ –T_12_), using primary antibodies against respiratory chain proteins subunits (ab110413). Lane 1 contains rat heart mitochondrial lysate as the positive control and lane 10 –rat muscle sample (m. gastrocnemius) as the internal standard.

After electrophoresis, proteins were transferred to nitrocellulose membranes (Amersham Hybond^™^, GE^™^ Healthcare, Pittsburgh, PA, USA) at a constant voltage (35 V) in transfer buffer at 4°C. For detection of the content of subunits of mitochondrial complexes, membranes were incubated overnight at 4°C with Total OXPHOS Rodent WB primary antibody cocktail (ab110413; Abcam, Cambridge, UK) containing 5 mouse monoclonal antibodies, one each against CI subunit NDUFB8 (ab110242), CII-30 kDa (ab14714), CIII-Core protein 2 (ab14745), CIV subunit I (ab14705) and CV alpha subunit (ab14748). At the same time, other membranes were incubated overnight at 4°C with a primary antibody specific to β-actin (ab8227; Abcam, Cambridge, UK). The membranes for the PGC-1α analysis were incubated 16 hours at 4°C (ab54481, Abcam). All membranes were then incubated with an HRP-conjugated secondary antibody. The protein bands were visualized using an enhanced chemiluminescence method (ECL Western Clarity, 170–5060; BioRad, Hercules, CA, USA), with GeneGnome 5 (GeneSys 1.2.7.0, Syngene Bio Imaging, Cambridge, UK) and GeneTools Syngene (Cambridge, UK) software used for densitometry analysis. Identification of the appropriate subunits of mitochondrial protein complexes in membranes was performed by comparing the analysed bands with those of the positive control (rat heart mitochondrial lysate), which was run in each gel (Fig 1). The values of optical density determined for each subunit of mitochondrial protein were normalized to the internal standard (rat muscle sample that was run on each gel) and then normalized also to β-actin (loading control). In case of PGC1-α protein level was normalized to the internal standard and then normalised to GAPDH (ab8245, Abcam, loading control). Data were presented as arbitrary units (a.u.).

### Statistical methods

The results are presented as means and standard deviations (SD). p-values describing significance of differences are also reported. Statistical analyses were performed using Statistica 12.0 software (StatSoft, Tulsa, OK, USA).

The basic contractile properties of the MUs was tested using the Shapiro-Wilk test (normality of distribution) and Levene’s test (equality of variances). None of the data met the requirements for parametric tests (the distribution was not normal, or a lack of homogeneity of variances was noted), therefore nonparametric Dunn’s post hoc test was used to compare individual groups.

Statistical analysis of a proportion of the three MU types was made with the Two Proportion Z-test. Changes in total body and muscle mass were analysed using the Shapiro-Wilk test (normality of distribution) and Levene’s test (equality of variances), and a Tukey’s HSD post-hoc test was used to determine between-group differences.

In order to analyse the impact of training on mitochondrial DNA copy number relative to nuclear DNA (mtDNA/nDNA) in MGS and MGF over the time course of training, the data were analysed using one-way ANOVA with four groups (C, 2W, 4W and 8W). Standard ANOVA assumptions were satisfied in this case. The basic questions to be answered from analysis of the empirical data were:

1. What was the long-term effect (if any) of training, observed after consecutive weeks?
2. Were any individual differences statistically significant?

In the first stage of the analysis, we used planned comparisons with polynomial contrasts, with the training groups ordered consecutively by training time. We detected highly significant (even after Bonferroni correction) and positive linear contrasts for mtDNA/nDNA data (p = 0.001 and p = 0.023 for MGF and MGS, respectively). No other contrasts were found to be significant. Having detected an overall increasing trend for the training groups, we then checked the significance of the differences with respect to the control, using Dunnett’s post hoc test with one-sided p-values. Moreover, the difference in mitochondrial DNA copy number between the MGF and MGS was tested using a Student’s t-test.

In order to analyse the impact of training on *Myh7*, *Atp2a2*, *Pvalb*, *Mfn1*, *Mfn2*, *Opa1* gene expression levels and on the electron transport chain protein complexes of the mitochondria over the training time course in both parts of the MG (MGS and MGF), the data were analysed using one-way ANOVA and then the significance was checked using Dunnett’s post hoc test and one-sided p-values are presented. The training-induced changes in content of PGC-1α in MGS have also been analysed using one-way ANOVA and Dunnett’s post hoc test. In case of some data (genes expressions), the tests detected possible departures from the assumptions of homoscedasticity. Although, it is well known that ANOVA procedures are relatively robust against violating this assumption (cf., e.g., [55]), as a cross-check, we also analyzed the data using multiple comparisons tests of Games-Howell and Dunnett in versions that do not assume homogeneous variances, as implemented in R-packages userfriendlyscience (function one-way) and multcomp (function glht)–see: https://cran.r-project.org/. The results were exactly the same in the sense of the differences being statistically significant or insignificant in exactly the same cases, so we report only the results of the standard ANOVA.

## Results

### Body and muscle mass

The mean body mass of trained rats was lower in the 4W (p = 0.03; Levene’s test, Tukey’s HSD post-hoc test) and 8W (p = 0.01; Levene’s test, Tukey’s HSD post-hoc test) groups compared to the control animals. However, the mean mass of MG muscle and the muscle-to-body ratio were not significantly different between groups (S2 File).

### Proportion of motor units

**Fig 2:**
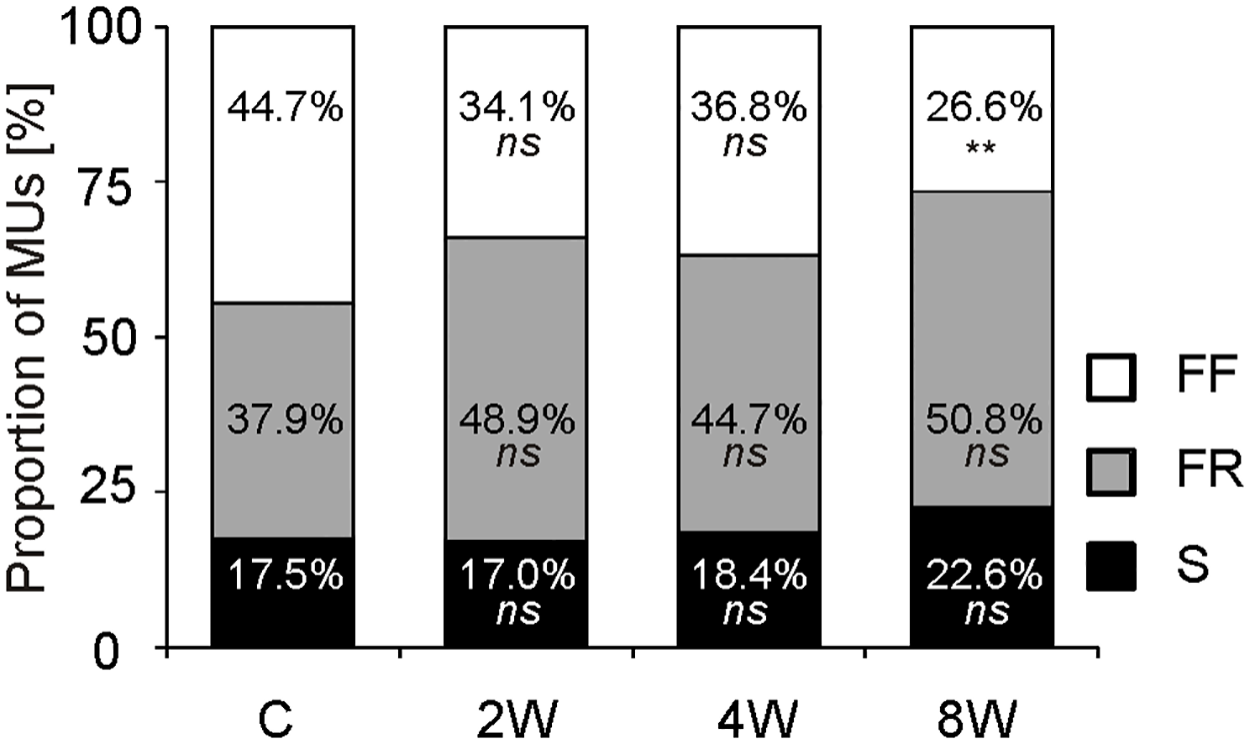
Distribution of three types of motor units in control and trained animals. Distribution of the three motor unit types of the medial gastrocnemius muscle in the sedentary control group (C) and those trained for 2 weeks (2W), 4 weeks (4W) and 8 weeks (8W). Fast fatigable (FF) MUs are represented in white, fast fatigue resistant (FR) MUs in grey, and slow (S) MUs in black. Two-sided p-value represents the statistical significance of the training period (2, 4 or 8 weeks) with respect to the control group (Two Proportion Z-test). The symbols * denote values significantly different: * p < 0.05, ** p < 0.01, *** p < 0.001 (S3 File).

The contractile properties of 103 MUs in the C group, 135 MUs in the 2W group, 114 MUs in 4W group and 124 MUs in 8W group were investigated. Training modified the proportion of FF, FR and S MUs in the studied groups (Fig 2). There was a trend toward a decreased proportion of FF MUs observed with increasing training duration, although a statistically significant difference was only detected between the 8W and C groups (p = 0.004). The opposite trend was noted for the FR-type MUs (the difference between 8W and C group at p = 0.05).

### Contractile properties of motor units

**Fig 3:**
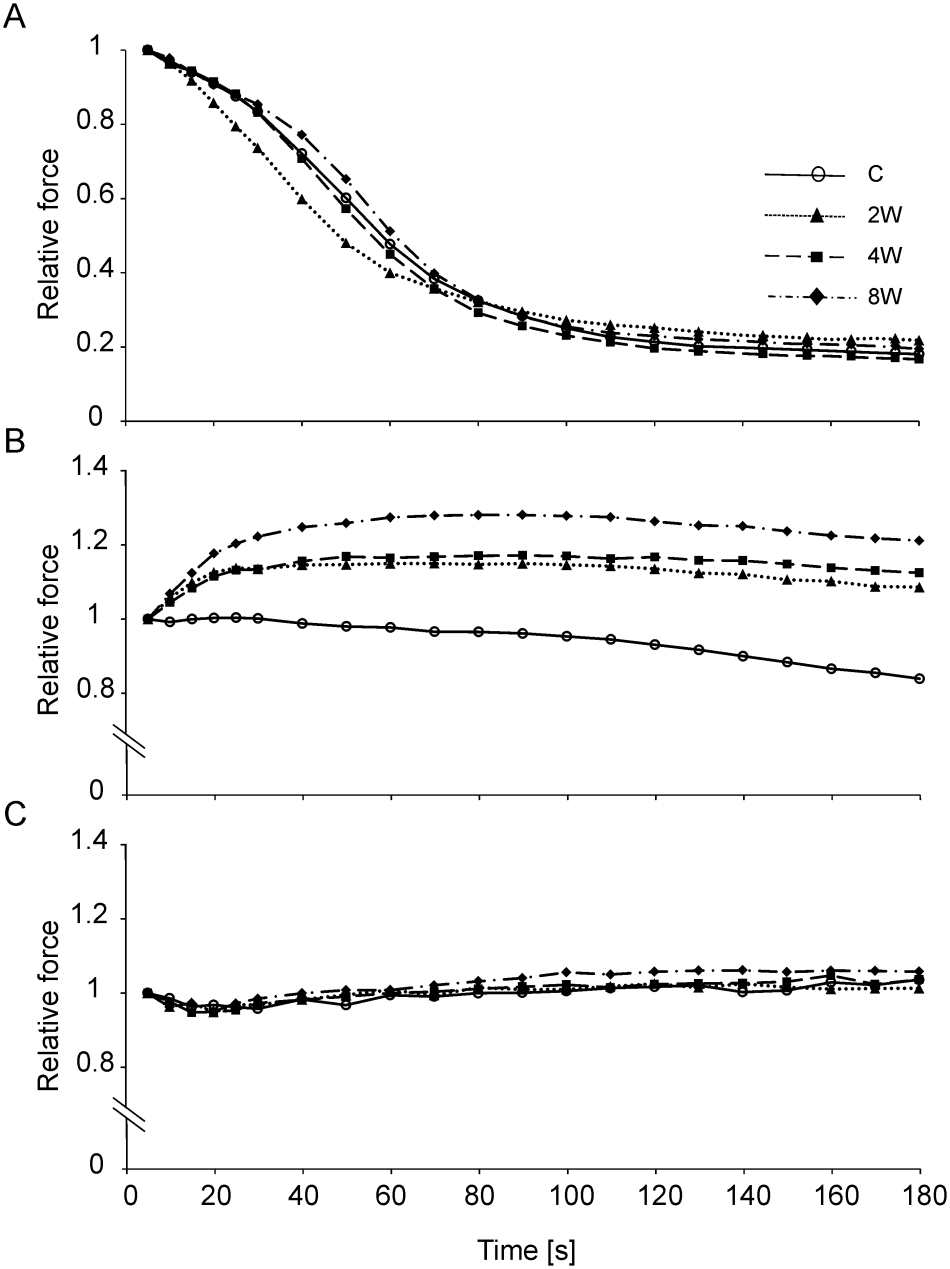
Force changes during the fatigue test for motor units in control and trained animal. The profiles of the fatigue test for fast fatigable (FF; panel A), fast fatigue-resistant (FR; panel B) and slow (S; panel C) motor units. The control (C) group is represented by open circles, the group trained for 2 weeks (2W) is represented by filled triangles, the group trained for 4 weeks (4W) is represented by filled squares, and the group trained for 8 weeks (8W) is represented by filled diamonds. The mean force over time for each group is presented as the relative values reached during the fifth second of the fatigue test, accepted as 1.0 (S5 File).

The endurance training influenced the basic contractile properties of the MUs, mainly of the FR-type. Differences between the C group and the 2W, 4W and 8W groups were significant, including the shorter contraction time (p = 0.02, p = 0.001 and p = 0.006 for 2, 4 and 8 weeks, respectively) and the half-relaxation time (p = 0.009, p = 0.0008 and p = 0.008 for 2, 4 and 8 weeks, respectively), the lower twitch-to-tetanus ratio (p = 0.007, p = 0.0004 and p < 10^−4^ for 2, 4 and 8 weeks, respectively) and the higher fatigue index (p = 0.009, p = 0.03 and p = 0.001 for 2, 4 and 8 weeks, respectively). Additionally, the twitch-to-tetanus ratio in the 8W group was significantly lower when compared to the 2W group (p = 0.006) (Table 1). The increased fatigue resistance of FR MUs in the trained animals was reflected not only by a higher fatigue index, but also by differences in the course of the fatigue test. For FR MUs of the trained groups, the curves representing the percentage change in tetanus force during the test were plotted above control values (Fig 3B). This was due to an increased ability to potentiate the force at the beginning of activity in trained animals. The force of tetanic contractions in the fatigue test increased by 9.08 ± 9.63%, 26.58 ± 20.93% (p = 0.0001), 32.14 ± 29.8% (p = 0.0001), and 38.81 ± 40.16% (p < 10^−4^), for the C, 2W, 4W and 8W groups, respectively (for a comparison of C to three experimental groups Dunn’s post hoc test was used) (S4 File).

**Table 1:** Contractile properties of the studied motor units in control and trained animals.

	CT [ms]	HRT [ms]	TwF [mN]	TetF [mN]	Tw/Tet	PTP	FatI
FF							
C	15.8 ± 3.0	21.3 ± 3.6	114.7 ± 34.7	289.0 ± 64.1	0.38 ± 0.07	1.33 ± 0.19	0.20 ± 0.11
2W	14.7 ± 2.6	19.9 ± 2.9	93.9 ± 39.1 *	243.8 ± 69.5 *	0.37 ± 0.09	1.33 ± 0.19	0.23 ± 0.14
4W	15.7 ± 3.4	21.5 ± 3.7	112.4 ± 44.4	295.0 ± 85.2 †	0.37 ± 0.10	1.34 ± 0.24	0.19 ± 0.10
8W	15.3 ± 2.1	20.9 ± 2.5	96.6 ± 40.2	251.5 ± 77.1	0.38 ± 0.09	1.35 ± 0.24	0.22 ± 0.13
FR							
C	16.5 ± 2.6	21.7 ± 3.0	32.3 ± 20.1	119.9 ± 58.4	0.25 ± 0.06	1.39 ± 0.17	0.86 ± 0.12
2W	14.9 ± 2.1 *	19.8 ± 2.5 **	27.7 ± 17.7	121.6 ± 56.5	0.21 ± 0.06 **	1.39 ± 0.23	0.93 ± 0.09 **
4W	14.5 ± 2.7 **	19.1 ± 3.1 ***	26.4 ± 17.0	122.4 ± 56.0	0.20 ± 0.07 ***	1.43 ± 0.26	0.93 ± 0.10 *
8W	14.7 ± 2.4 **	19.8 ± 2.7 **	25.5 ± 19.1	134.0 ± 68.9	0.18 ± 0.05 ***††	1.36 ± 0.19	0.94 ± 0.09 **
S							
C	26.2 ± 4.5	29.9 ± 4.1	5.0 ± 1.9	39.3 ± 14.7	0.13 ± 0.05	0.97 ± 0.09	0.98 ± 0.05
2W	24.0 ± 3.2	27.4 ± 4.1	4.3 ± 1.6	36.3 ± 13.0	0.12 ± 0.02	0.98 ± 0.11	0.99 ± 0.04
4W	26.9 ± 5.5	31.5 ± 4.8	4.3 ± 2.0	35.3 ± 10.8	0.12 ± 0.04	0.94 ± 0.09	0.99 ± 0.03
8W	27.1 ± 5.9	29.6 ± 4.9	3.6 ± 1.3	36.3 ± 8.2	0.10 ± 0.02 **§	0.94 ± 0.11	1.00 ± 0.04

The mean values of the studied properties (± SD) of fast fatigable (FF), fast fatigue resistant (FR) and slow (S) motor units (MUs) in the control (C) group and the three trained groups, consisting of rats trained for 2 weeks (2W), 4 weeks (4W) and 8 weeks (8W).

CT, the contraction time; HRT, the half-relaxation time; TwF, the twitch force; TetF, the maximal tetanic force; Tw/Tet, the twitch-to-tetanus ratio; PTP, the post-tetanic potentiation; FatI, the fatigue index.

Statistical significance in comparison to the control (C) group is represented by * p < 0.05, ** p < 0.01, *** p < 0.001.

Statistical significance in comparison to the group trained for 2 weeks (2W) is represented by † p < 0.05, †† p < 0.01.

Statistical significance in comparison to the group trained for 4 weeks (4W) is represented by § p < 0.05 (Dunn’s post hoc test) (S1 File).

No significant changes in the course of the fatigue test were noted for FF and S MUs (Fig 3A and 3C). For FF MUs, changes in the contractile properties were only observed in the 2W group, which involved the lower twitch force when compared to the C group (p = 0.05) and the lower tetanus forces in comparison to the C group (p = 0.03) and the 4W group (p = 0.01). The S MUs were least affected by the endurance training, with significant changes only in the twitch-to-tetanus ratio of the 8W group, which was lower in comparison to the C group (p = 0.008) and the 4W group (p = 0.04) (Table 1).

### Force-frequency curve

**Fig 4:**
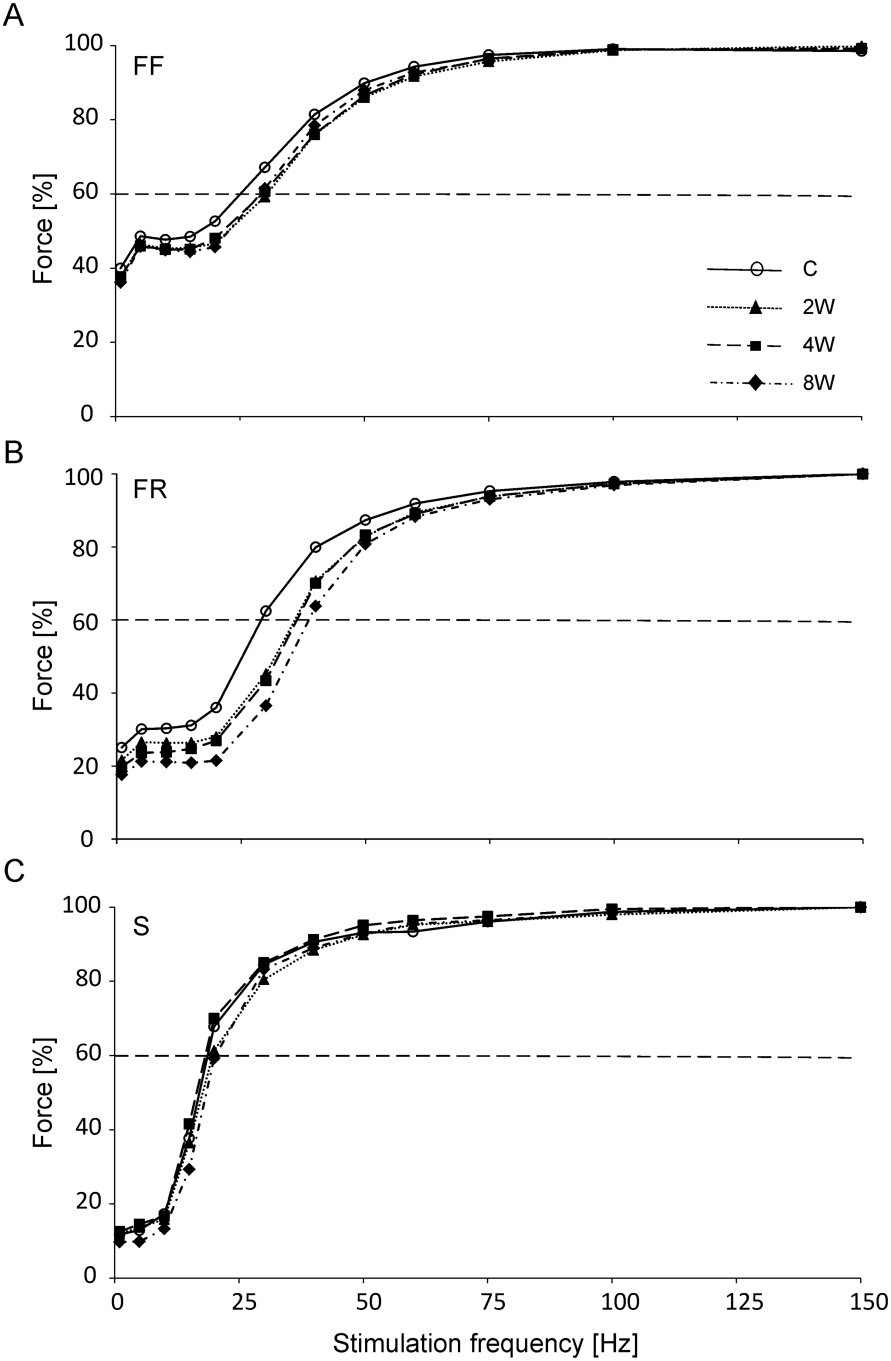
Force-frequency relationships of the three motor unit types in control and trained animals. Fast fatigable (FF) motor units (MUs) are presented in panel A, fast fatigue resistant (FR) MUs in panel B, and slow (S) MUs in panel C. The control (C) group is represented by open circles, the group trained for 2 weeks (2W) is represented by filled triangles, the group trained for 4 weeks (4W) is represented by filled squares, and the group trained for 8 weeks (8W) is represented by filled diamonds. The dashed horizontal lines in panels A–C indicate the level of 60% of the maximum force (normalized to the maximum tetanus force obtained at 150 Hz) (S6 File).

For FR MUs, the steep parts of force-frequency relationships of all experimental groups were shifted to the right when compared to the sedentary group (Fig 4B). These shifts were due to the requirement for higher stimulation frequencies in order to reach 60% of the maximum force (p < 10^−4^, p < 10^−4^ and p < 10^−4^ for 2, 4 and 8 weeks, respectively). Value of the 60% of the maximum force in 8W group was also significantly higher when compared to 2W group (p = 0.04) (Table 2).

**Table 2:** Properties of the force-frequency relationship in studied motor units in control and trained animals.

	C	2W	4W	8W
FF				
60%Fmax [Hz]	27.70 ± 6.37	29.81 ± 8.29	29.49 ± 9.84	29.05 ± 7.95
Slope [%Fmax 1Hz^-1^]	1.89 ± 0.46	1.84 ± 0.50	2.13 ± 0.79	2.05 ± 0.69
FR				
60%Fmax [Hz]	29.32 ± 4.62	35.29 ± 5.10 ***	35.49 ± 6.10 ***	38.21 ± 5.43 ***†
Slope [%Fmax 1Hz^-1^]	2.73 ± 0.70	2.71 ± 0.61	2.79 ± 0.65	2.99 ± 0.66
S				
60%Fmax [Hz]	18.83 ± 2.05	21.12 ± 5.63	18.54 ± 4.96	20.65 ± 3.16 §
Slope [%Fmax 1Hz^-1^]	5.35 ± 1.97	4.63 ± 2.50	6.87 ± 2.14 ††	4.98 ± 1.99 §

Values of parameters describing the force-frequency relationships (mean values ± SD) of the three basic types of motor units, fast fatigable (FF), fast fatigue resistant (FR) and slow (S), for the control (C) group and the three trained groups, consisting of rats trained for 2 weeks (2W), 4 weeks (4W) and 8 weeks (8W).

60%Fmax, the stimulation frequency necessary to reach 60% of the maximum force; slope, the slope of a steep part of the curve at approximately 60% of the maximum force.

Statistical significance relative to the control group is represented by *** p < 0.001.

Statistical significance relative to the group trained for 2 weeks (2W) is represented by † p < 0.05, †† p < 0.01.

Statistical significance in comparison to the group trained for 4 weeks (4W) is represented by § p < 0.05 (Dunn’s post hoc test) (S7 File).

The force-frequency curve for FF MUs in all trained groups did not differ in relation to the C group (Fig 4A), and the frequencies at 60% of the maximum force and slope of the curve were not affected by training (Table 2).

For S type units the force frequency curves in all trained groups did not differ in relation to the C group (Fig 4C and Table 2), however, the differences between experimental groups were noted, i.e. 60% of the maximum force in the 8W group was higher in comparison to the 4W group (p = 0.03) and the slope in the 4W group was higher both for 2W (p = 0.01) and for the 8W group (p = 0.03) (Table 2).

### Optimal tetanic contraction

Analysis of the optimal contraction of the MU in terms of contractile output of the four groups of animals, characterized by the maximum FTA per pulse, is presented in Table 3. The largest change in optimal tetanus occurred in FR MUs. Although the values of FTA per pulse were similar for all studied groups, the optimal tetanus was induced at a higher stimulation frequency for all trained groups (p = 0.0001, p = 0.0005 and p < 10^−4^ for 2, 4 and 8 weeks, respectively), which is directly related to shortening of the twitch time parameters (see section “Contractile properties of motor units” and Table 1). Moreover, lower values of the FTA per pulse were noted for FF MUs of the 2W group in comparison to the C group (p = 0.002), reflecting lower values of force and shortening of the twitch time parameters in this group (see section “Contractile properties of motor units” and Table 1).

**Table 3:** The optimal tetanus in studied motor units in trained and control animals.

	FTA per pulse [mN ms]	Frequency [Hz]	Relative force [%]
FF			
C	6486.7 ± 2141.4	31.7 ± 9.9	69.8 ± 7.6
2W	4820.6 ± 1788.7 **	37.8 ± 11.2	72.7 ± 8.3
4W	5989.0 ± 2288.8	34.9 ± 10.3	71.6 ± 7.3
8W	5201.7 ± 1768.9	36.4 ± 9.0	72.9 ± 8.3
FR			
C	2218.2 ± 1093.3	40.5 ± 7.2	82.7 ± 7.2
2W	2111.9 ± 1070.1	48.4 ± 8.5 ***	81.6 ± 6.6
4W	2041.9 ± 1014.7	48.0 ± 7.6 ***	82.7 ± 7.9
8W	2279.4 ± 1184.1	50.8 ± 8.3 ***	82.0 ± 7.3
S			
C	1385.1 ± 437.4	22.3 ± 5.0	75.9 ± 10.7
2W	1083.1 ± 426.7	22.6 ± 5.4	73.4 ± 13.3
4W	1217.2 ± 433.6	20.6 ± 3.9	74.7 ± 8.6
8W	1109.6 ± 348.1	25.2 ± 5.9	74.7 ± 9.8

Parameters of the optimal tetanus force (mean values ± SD) of the three basic types of motor units, fast fatigable (FF), fast fatigue resistant (FR) and slow (S), for the control (C) group and the three trained groups, consisting of rats trained for 2 weeks (2W), 4 weeks (4W) and 8 weeks (8W).

FTA per pulse, the force-time area per pulse; frequency, stimulation frequency of the optimal tetanus; relative force, the peak force of the optimal tetanus, expressed as a percentage of the maximum force. Statistical significance in relation to the control group is represented by ** p < 0.01 and *** p < 0.001 (Dunn’s post hoc test) (S8 File).

### Expression of myosin heavy chain type I (*Myh7*) and Ca^2+^-handling genes (*Atp2a2* and *Pvalb*) in the fast and in the slow part of the medial gastrocnemius

**Fig 5:**
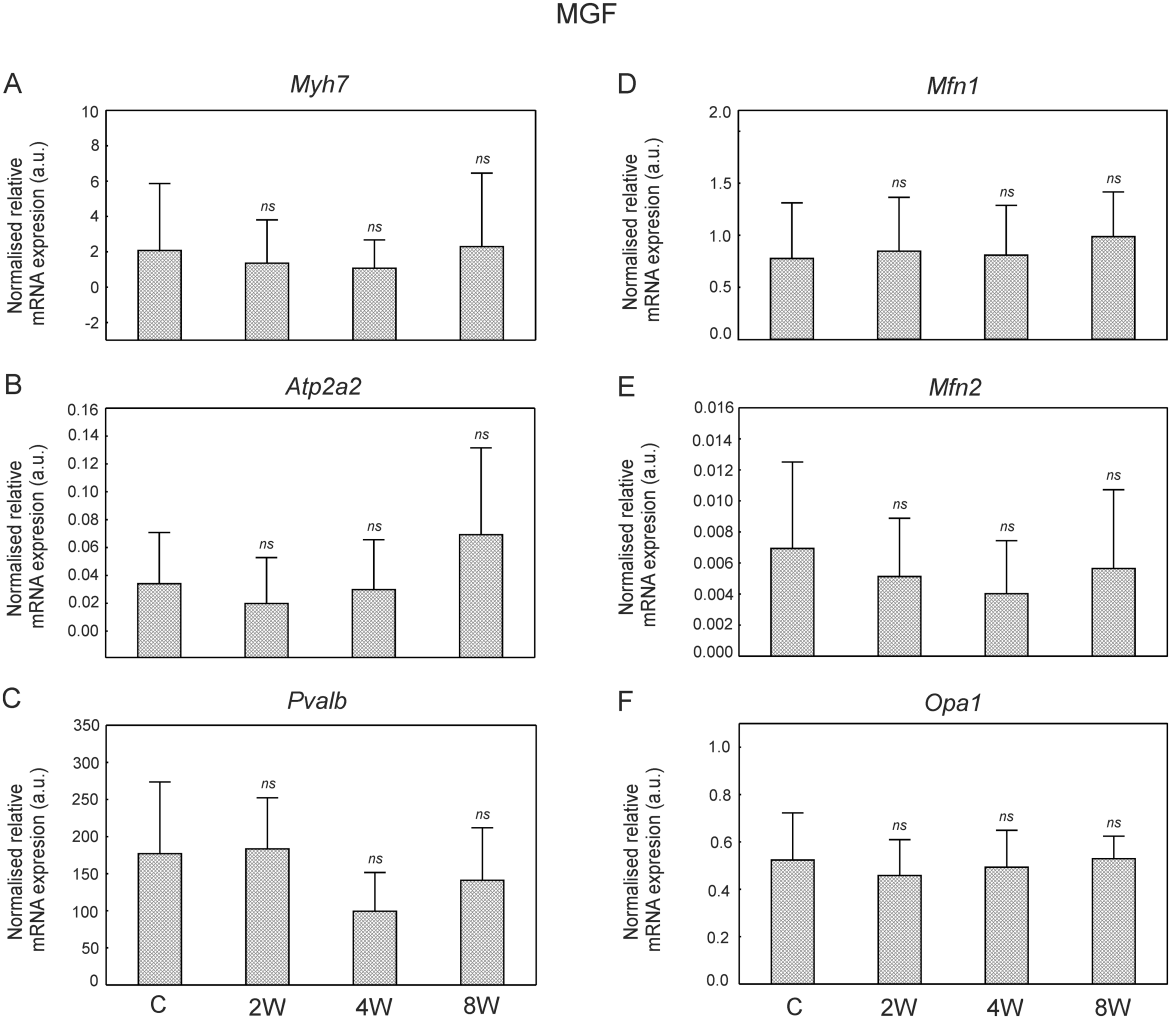
Relative mRNA expression levels in the MGF in control and trained animals. Relative mRNA expression of myosin heavy chain slow isoform (*Myh7*, panel A), SERCA2 (*Atp2a2*, panel B), parvalbumin (*Pvalb*, panel C), mitofusin 1 (*Mfn1*, panel D), mitofusin 2 (*Mfn2*, panel E), mitochondrial dynamin-like GTPase (*Opa1*, panel F) in the fast part of the medial gastrocnemius (MGF) after 2, 4 and 8 weeks of endurance training. Data are presented as mean + SD and normalized by the reference gene (*Hprt1*). One-sided p-value represents the statistical significance of the training period (2, 4 or 8 weeks) with respect to the control group (Dunnett’s post hoc test) (S9 File). The symbols * denote values significantly different: * p < 0.05, ** p < 0.01, *** p < 0.001.

No statistically significant changes were observed in the mRNA abundance levels of the slow isoform of myosin heavy chain (*Myh7)*, parvalbumin (*Pvalb*) and SERCA2 (*Atp2a2*) of MGF during the training period (2, 4 and 8 weeks) when compared to the control group (Fig 5A–5C).

**Fig 6:**
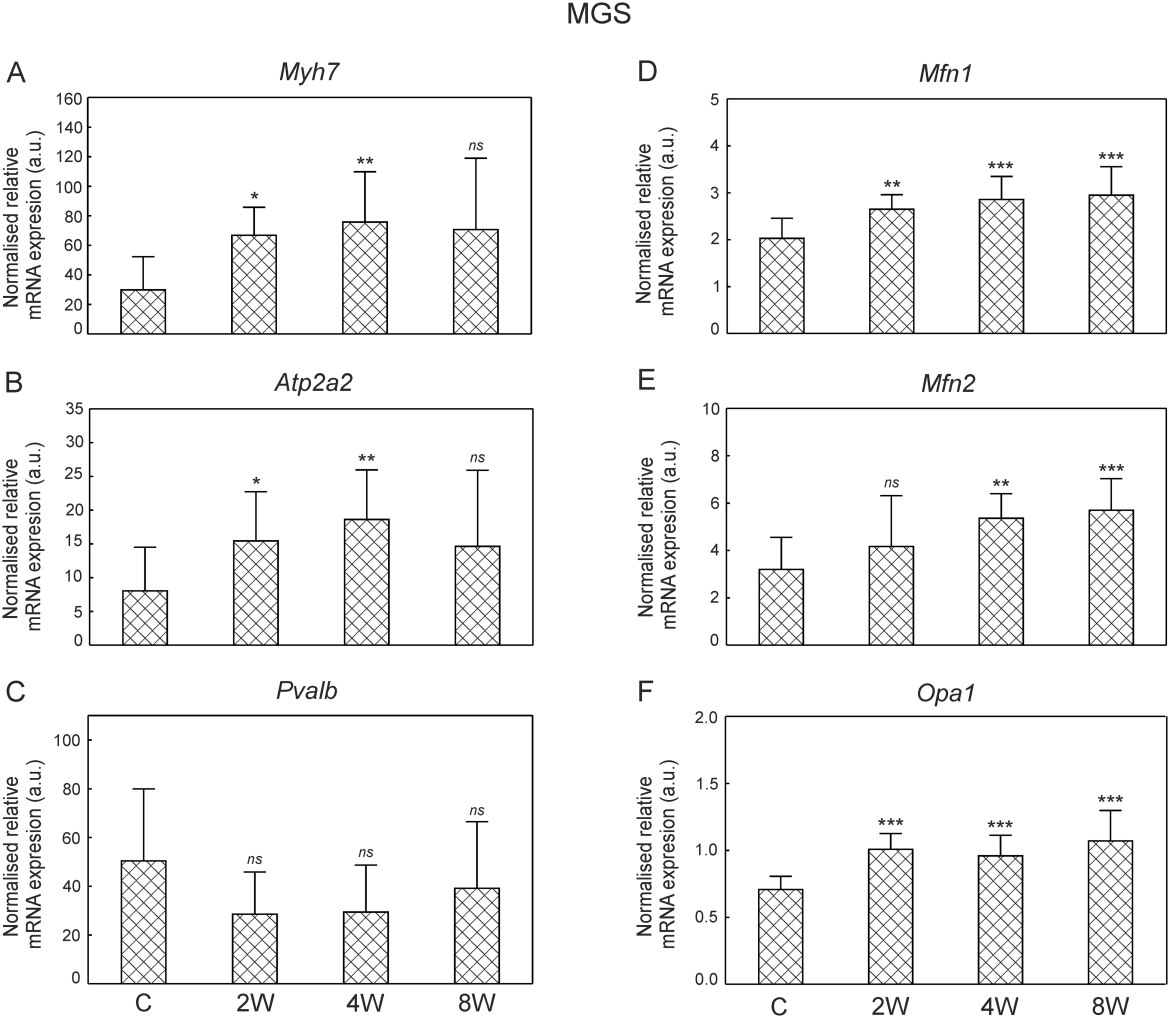
Relative mRNA expression levels in the MGS in control and trained animals. Relative mRNA expression of myosin heavy chain slow isoform (*Myh7*, panel A), SERCA2 (*Atp2a2*, panel B), parvalbumin (*Pvalb*, panel C), mitofusin 1 (*Mfn1*, panel D), mitofusin 2 (*Mfn2*, panel E), mitochondrial dynamin-like GTPase (*Opa1*, panel F) in the slow part of the medial gastrocnemius (MGS) after 2, 4 and 8 weeks of endurance training. Data are presented as mean + SD normalized by the reference gene (*Hprt1*). One-sided p-value represents the statistical significance of the training period (2, 4 or 8 weeks) with respect to the control group (Dunnett’s post hoc test) (S10 File). The symbols * denote values significantly different: * p < 0.05, ** p < 0.01, *** p < 0.001.

In the slow part of the MG (MGS) a significantly greater *Myh7* mRNA level after 2 (p = 0.02) and 4 (p = 0.002) weeks of training was found when compared to the control group (Fig 6A). Moreover, in this part of MG significantly higher *Atp2a2* mRNA content was observed after 2 (p = 0.04) and 4 (p = 0.004) weeks of training when compared to the control group (Fig 6B). No changes in *Pvalb* mRNA were noticed during the training period (Fig 6C).

### Expression of mitofusin 1 (*Mfn1*), mitofusin 2 (*Mfn2*), dynamin-like protein (*Opa1*) in the fast and in the slow part of the medial gastrocnemius

No statistically significant changes were observed in the mRNA levels of mitofusin 1 (*Mfn1)*, mitofusin 2 (*Mfn2)*, mitochondrial dynamin-like GTPase (*Opa1*) in the MGF during the training period (2, 4 and 8 weeks) (Fig 5D–5F).

In the MGS a significantly higher *Mfn1* mRNA abundance level after 2 (p = 0.002), 4 (p < 10^−4^) and 8 (p < 10^−4^) weeks of training have been found when compared to the control group (Fig 6D), whereas a significantly higher *Mfn2* mRNA content has been observed after 4 (p = 0.003) and 8 weeks of training (p = 0.0006) when compared to the control group (Fig 6E). *Opa1* mRNA levels has been found to be significantly greater after 2 (p < 10^−4^), 4 (p = 0.001) and 8 (p < 10^−4^) weeks of training when compared to the control group (Fig 6F).

### Mitochondrial mtDNA copy number (mtDNA/nDNA) in the fast and in the slow part of the medial gastrocnemius

**Fig 7:**
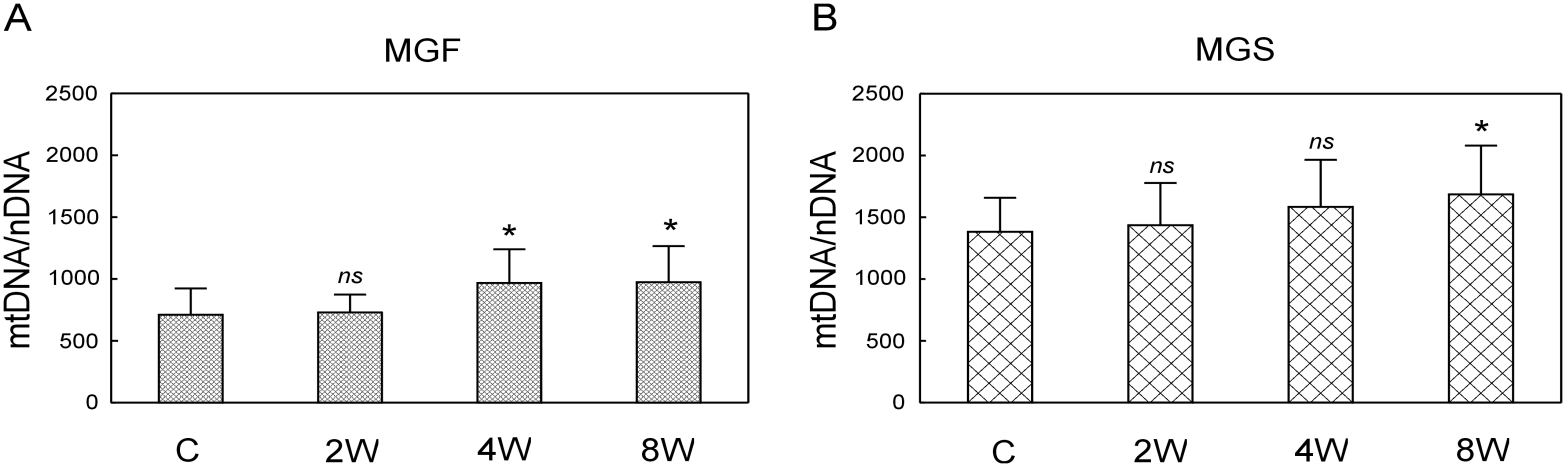
mtDNA copy number in the MGF and the MGS in control and trained animals. Mitochondrial DNA copy number (mtDNA/nDNA ratio) in the fast part of the medial gastrocnemius (MGF, panel A) and the slow part of the medial gastrocnemius (MGS, panel B) after 2, 4 and 8 weeks of endurance training. Data are presented as mean + SD. One-sided p-value represents the statistical significance of the training period (2, 4 or 8 weeks) with respect to the control group (Dunnett’s post hoc test) (S11 File). The symbols * denote values significantly different: * p < 0.05, ** p < 0.01, *** p < 0.001.

**Fig 8:**
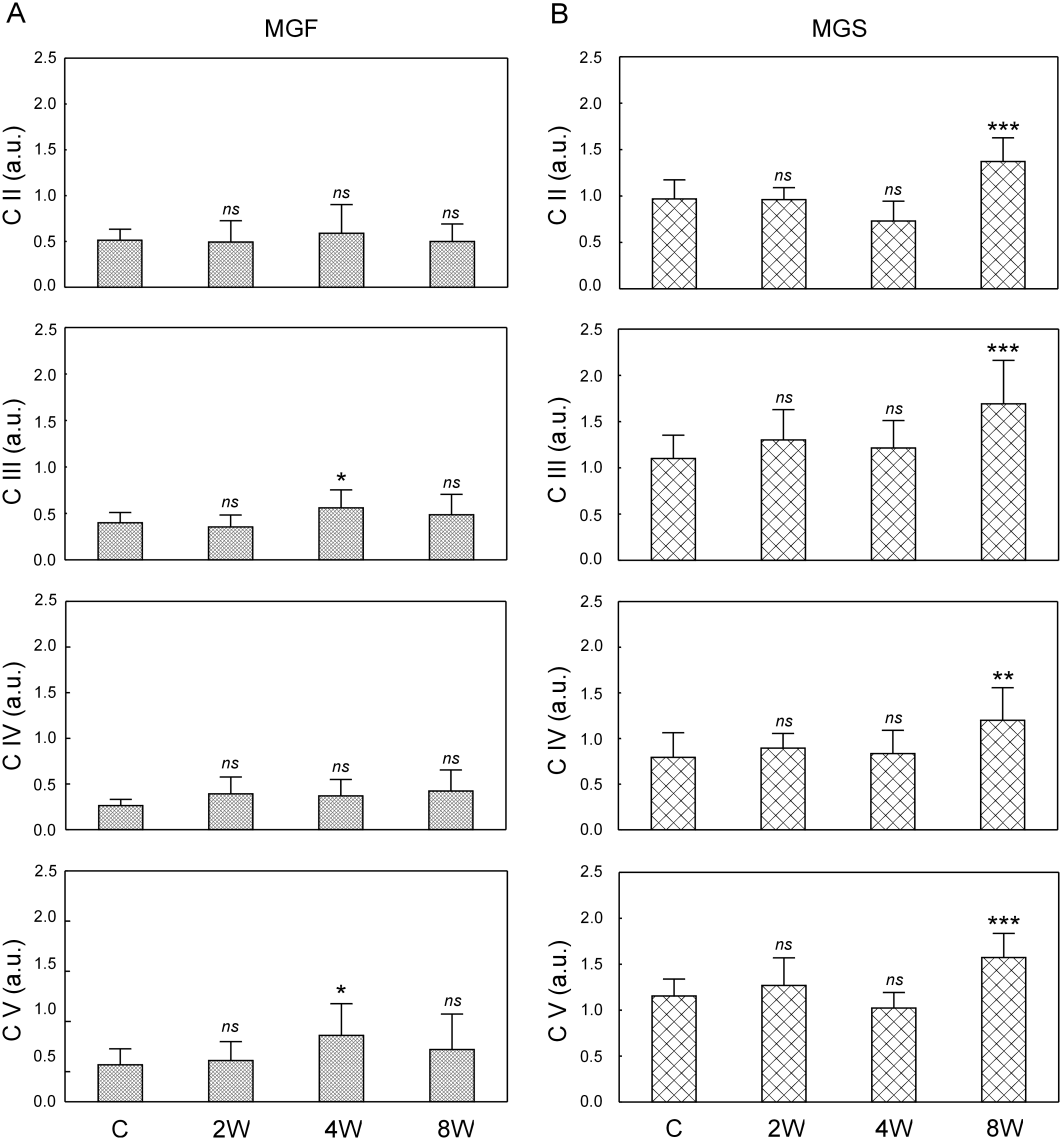
Mitochondrial protein ETC complexes in the MGF and the MGS in control and trained animals. Subunits of mitochondrial protein complexes (C II, C III, C IV, C V) in the fast part of the medial gastrocnemius muscle (MGF, panel A, S12 File) and in the slow part of the medial gastrocnemius (MGS, panel B, S13 File) after 2, 4 and 8 weeks of endurance training. Data are presented as mean + SD and normalized by the reference protein β-actin and the internal standard. One-sided p-value represents the statistical significance of the training period (2, 4 or 8 weeks) with respect to the control group (Dunnett’s post hoc test). The symbols * denote values significantly different: * p < 0.05, ** p < 0.01, *** p < 0.001.

The mtDNA/nDNA ratio in the MGF of the control group had a mitochondrial DNA copy number of 711 ± 213. Endurance training resulted in an overall increase in the mtDNA/nDNA ratio (p = 0.001). When compared to the control group, a significantly higher mtDNA/nDNA ratio was found after both 4 weeks (~36%, p = 0.01; Fig 7A) and 8 weeks (~37%, p = 0.01; Fig 7A) of training.

The mitochondrial DNA copy number in the MGS of the control group was estimated at 1382 ± 275, which was significantly higher (~2 times) than the mtDNA/nDNA ratio in MGF (711 ± 213, p = 0.0003). Endurance training resulted in an overall increase in the mtDNA/nDNA ratio (p = 0.023). A significantly higher mtDNA/nDNA ratio was observed after 8 weeks of training when compared to the sedentary group (~22%, p = 0.04; Fig 7B).

### Mitochondrial electron transport chain (ETC) protein complexes in the fast and in the slow part of the medial gastrocnemius

The effect of endurance training (2, 4 and 8 weeks of training) on the subunits of mitochondrial protein complexes, i.e. complex II (succinate dehydrogenase), complex III (cytochrome bc_1_ complex), complex IV (cytochrome-c oxidase) and complex V (ATP synthase), was analysed separately in the MGS and MGF (Fig 8). Complex I subunit NDUFB8 protein was not detected in any of the analysed muscle samples. Fig 1 shows a typical immunoblot of proteins extracted from the MGF.

In the MGF, no significant changes in the subunits of mitochondrial electron transport chain protein complexes were found except a significant, transient increase in C III subunit and C V subunit after 4 weeks of training when compared to the control group (Fig 8A).

In the MGS, a significant increase in all measured subunits of mitochondrial electron transport chain protein complexes were found after 8 weeks of training, whereas no significant changes were detected after 2 and 4 weeks of training (p > 0.05). Specifically, significantly higher content of complex II subunit (~30%, p = 0.0001), complex III subunit (~38%, p = 0.0006), complex IV (~58%, p = 0.001) and complex V subunit (~33%, p = 0.0002) were observed after 8 weeks of training when compared to the control group (Fig 8B).

### Peroxisome proliferator-activated receptor gamma co-activator (PGC-1α) protein in the slow part of the medial gastrocnemius

**Fig 9:**
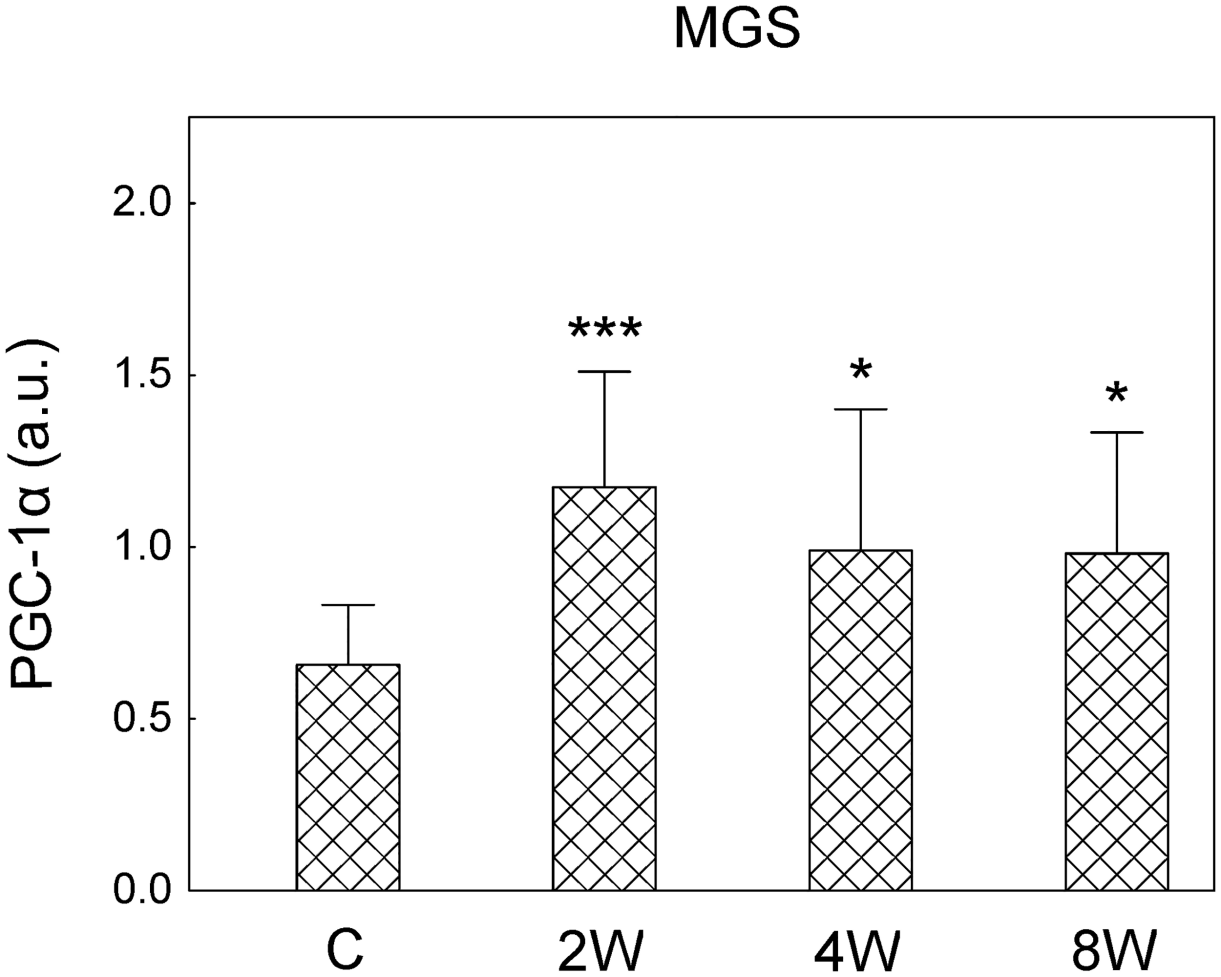
PGC-1α in the MGS in control and trained animals. PGC-1α in the slow part of the medial gastrocnemius (MGS, S14 File) after 2, 4 and 8 weeks of endurance training. Data are presented as mean + SD and normalized by the reference protein GAPDH and the internal standard. One-sided p-value represents the statistical significance of the training period (2, 4 or 8 weeks) with respect to the control group (Dunnett’s post hoc test). The symbols * denote values significantly different: * p < 0.05, ** p < 0.01, *** p < 0.001.

The effect of the endurance training (2, 4 and 8 weeks) on the PGC-1α has been analysed in the slow part of gastrocnemius (MGS). A significantly higher PGC-1α in the MGS has been found after 2 (~60%, p = 0.001), 4 (~40%, p = 0.02) and 8 weeks of training (~40%, p = 0.03) (Fig 9).

## Discussion

The present study has documented, for the first time, the adaptive plasticity of the contractile properties of MUs in the hind limb muscle (MG) during moderate to high intensity endurance training, and related these changes to the progression of mitochondrial biogenesis. Results of the present study indicate that functional changes in contractile properties in MG muscle including an increase in fatigue resistance, shortening of the twitch time parameters and an increase in ability to potentiate force are evident after 2 weeks of training (Fig 3 and Table 1) but are restricted to FR MUs. At this stage of training no significant changes in the mtDNA/nDNA and mitochondrial ETC protein contents have been found in either the MGF or MGS (Figs 7 and 8). Nevertheless, as early as after 2 weeks of training an increase in *Mfn1*, *Opa1* mRNA (Fig 6D and 6F) accompanied by an increase in PGC-1α content only in the slow part of gastrocnemius has been observed (Fig 9). Subsequently, at more advanced stage of training, particularly after 8 weeks, a clear intensification of mitochondrial biogenesis has been observed, as evidenced by an increase in the mitochondrial DNA copy number (Fig 7) and content of electron transport chain proteins (Fig 8).

### Time-dependent changes in motor unit contractile properties during endurance training

The most significant changes in contractile properties in response to endurance training observed in FR MUs concerned predominantly an increase in resistance to fatigue and a shortening of the contraction time. In addition, these adaptive changes became apparent very early during the training period, after only 2 weeks of increased activity (Table 1). It should also be noted that the altered activity led to an increased proportion of the FR units, parallel to a decreased proportion of FF MUs, most likely due to the physiological transformation of FF to FR units (Fig 2). As the running speed applied in the training protocol was lower than the maximum running speed recorded for rats (75 m min^-1^) [56], this suggests that a portion of the FF MUs were not involved in the activity, and therefore, their contractile properties remained unchanged. Therefore, adaptations in the contractile properties in response to training were limited to this group of MUs, activity of which was sufficiently altered. It should also be stressed that, in general, the contractile properties of S MUs were not modified, probably because the training did not lead to a considerably increase of the normal activity level of S MUs; according to Hennig and Lømo [57] S MUs of rat soleus contract for 5–8 hours during normal daily activity.

A shortening of the twitch time parameters of FR MUs, observed in this study, is in agreement with results of previous experiments investigating several other types of increased motor activity, such as low-intensity treadmill training with a locomotion speed of 16.2 m min^-1^ [31], muscle functional overload induced by a tenotomy of synergists [58], or weight-lifting training [29]. On the other hand, this finding is in contrast to the prolonged twitch reported in rats subjected to the total transection [30] or a hemisection of the spinal cord [26]. Therefore, one may conclude that various forms of increased activity result in a twitch shortening. The twitch shortening reported in the current study influenced the force-frequency relationship of the FR MUs in the groups of trained animals showing a lower levels of force at the respective stimulation frequencies in comparison to control. This suggests that the motoneurons of trained animals needed to increase their discharge frequencies to efficiently activate the muscle fibres. There are, however, considerable discrepancies concerning the MU firing rate in studies that have compared athletes to control individuals. Some studies have revealed no changes in the mean MU firing rate after 4 weeks of endurance training [32], however, Vila-Chã et al. [2] reported a lower MU firing rate after 6 weeks of cycle ergometer training, and conversely, a higher MU firing rate after strength training. The results of studies by Beaumont and Gardiner [36,37] clearly showed that increased neuromuscular activity, due to spontaneous running exercise or endurance treadmill training, influenced the basic biophysical properties of motoneurones (mainly the resting membrane potential and the spike trigger level). These changes reflect adaptive alterations in ion conductance, which helps to offset the decreased membrane excitability which occurs during sustained excitation.

The ability to potentiate the force during activity of the FR MUs increased progressively with the training duration, starting from the 2W group (Fig 3). The largest potentiation was noted in the 8W group. Authors of a recent review [59] concluded that the primary mechanism underlying force potentiation is the phosphorylation of myosin (stimulation-induced elevation in regulatory light chain phosphate incorporation by skeletal myosin light chain kinase). However, they also indicated a secondary mechanism for this force modulation occurring in some muscle fibres, a stimulation-induced elevation in resting Ca^2+^. Force potentiation is a phenomenon that is dependent on numerous factors: species, muscle fibre type, stimulus frequency and duration, temperature, muscle length, and type of contraction [59]. All these variables were carefully controlled in the electrophysiological experiments described in the current study, therefore, the mechanisms responsible for increased potentiation were likely direct results of the training. Vanderboom et al. [59] suggested that regulatory light chain phosphorylation offsets the fatigue, which is in agreement with the higher fatigue index of FR MUs noted in our study. Potentiation has an important physiological role, not only during isometric contraction, but also in dynamic force, and therefore, plays an important role in muscle function. It is also worth noting that peripheral diseases negatively influence the ability of forces to potentiate [60–62]. Therefore, it seems possible to conclude that, increased or decreased activity affects differently force potentiation of fast muscle fibres.

### Time-dependent changes in the expression of *Myh7* and *ATP2a2* during the endurance training

The above reported by us functional changes in MUs properties, observed predominantly in FR MUs as early as after 2 weeks of training, such as an increase in resistance to fatigue, were accompanied by a transient significant increase in the expression of slow myosin heavy chain mRNA and Ca^2+^ ATPase 2 mRNA in MGS (Fig 6A and 6B). These results show that fast-to-slow transition at mRNA level occurs at the early stage of training and takes place in the slow part of the muscle (MGS, Fig 6A and 6B) with higher content of fatigue resistant MUs [39,41]. No effect of training on the parvalbumin expression both in the fast and slow parts of gastrocnemius has been observed (Figs 5C and 6C). One should be aware of the fact, that as reported earlier the transformation of muscle fibre at protein level (mainly MyHC transition) is rather a slow process and usually requires several weeks of training [9,10]. Therefore, the phenomenon of an increase in fatigue resistance in the early stage of training observed in the present study, cannot be directly dependent upon myosin heavy chain protein transition, despite the presence of the signal of the transformation at mRNA level.

### Time-dependent changes in mitochondrial network reorganisation and mitochondrial biogenesis during the endurance training

In the present study, we have found that endurance training in its early stage (just after 2 weeks) increases expression of GTPases involved in mitochondrial remodeling such as mitofusin 1, which controls outer mitochondrial membrane fusion (*Mfn1*, Fig 6D) and dynamin-like protein (*Opa1*, Fig 6F) governing the inner mitochondrial membrane fusion and cristae remodeling [63]. Moreover, an increase in the master regulator of mitochondrial biogenesis PGC-1α has been observed as early as after 2 weeks of training (Fig 9). These changes however, were limited to the MGS. At this early stage of training no significant changes in mtDNA copy number and ETC proteins content have been found both in the MGS and in the MGF (Figs 7 and 8).

The training-induced signals for reorganization of the skeletal muscle mitochondrial network were present from the beginning of endurance training, but only in the MGS (Fig 6D and 6F), which is the part of muscle mostly affected by the applied training program possessing higher (about 2-times) mitochondria content than the MGF (p = 0.0003). Moreover, an increase in *Opa1* mRNA as early as after 2 weeks of training (Fig 6F) shows that the mitochondrial cristae remodeling start from the beginning of endurance training. These results clearly show that the applied training program induces mitochondrial rearrangement processes [64,65]. Opa1-dependent cristae remodeling during endurance training seems to be important for the maintenance of the proper functionality of the oxidative phosphorylation (OHPHOS), since it has been found that cristae shape affects the performance of the electron transport chain complexes [63]. Interestingly, recently Nielsen et al. [66] have observed higher cristae density in the mitochondria of endurance-trained athletes when compared to recreationally trained people. These authors have proposed that increasing mitochondrial inner membrane surface compromises an alternative mechanism to mitochondrial volume increase for an enhancement of energy production after endurance training [66].

As found in the present study, the signals for mitochondrial network reorganization in the MGS (Fig 6) and an increase in PGC-1α content in this part of muscle precede an increase in the mitochondrial electron transport chain proteins content (Fig 8) and mtDNA copy number (Fig 7). Intensified mitochondrial biogenesis after endurance training is one of the most important adaptive responses leading to enhanced muscle energy homeostasis during exercise, as well as resistance to fatigue [1,4,5,11,16,19]. The commonly used expression “training-induced increase in mitochondrial biogenesis” is very vague and could be a source of confusion, as argued before [12]. Namely, the term “an increase in mitochondrial biogenesis” in the literature is associated often with several markers including e.g. mtDNA copy number, mitochondrial enzymes activity and content (e.g., citrate synthase and cytochrome-c oxidase) as well as proteins engaged in the regulation of this process such as PGC-1α [12,18,67]. However as pointed by Zoladz et al. [12] not the structural, but rather functional markers, such as cytochrome-c oxidase activity determining muscle OXPHOS capacity, should be predominantly considered. This also raises the relevant question of which markers of mitochondrial biogenesis are the most significant in terms of resistance to fatigue. It has recently been argued that the best marker of a training-induced increase in OXPHOS, leading to enhanced muscle metabolic stability, is an increase in cytochrome-c oxidase activity [12]. In the present study we did not measure the activity of cytochrome-c oxidase, however we found a significant increase in the content of subunit I of cytochrome-c oxidase (CIV complex, Fig 8B) as well as an increase in the content of other subunits of mitochondrial ETC complexes no sooner than after 8 weeks of training, in view of no changes in this protein in MGF during the training period (Fig 8A). An increase in mitochondrial ETC protein content after 8 weeks of training in the MGS was preceded by an increase in the PGC-1α found as early as after 2 weeks of training. The elevated PGC-1α in the MGS has been observed also after 4 and 8 weeks of training (Fig 9). This result is in agreement with our previous study showing that 8 weeks of endurance training up-regulates mitochondrial biogenesis markers such as Nrf2, PGC-1α as well as proteins involved in fatty acid metabolism [68]. On the other hand, an increase in mtDNA copy number was found at the final stage of training (8 weeks) in both the MGF and MGS (Fig 7). This suggests that while mitochondrial biogenesis, as evidenced by an increase in mtDNA copy number, is present in the slow and in the fast parts of this muscle, the enhancement of muscle oxidative capacity (as judged based on the increase in cytochrome-c-oxidase content) after training is most likely increased only in the MGS (Fig 8B).

It should be highlighted that in this study we used endurance training of a moderate to high intensity, with a gradual increase in training workload over 8 weeks, as originally described by Dudley et al. [38] (for details see “Materials and methods” section). This scenario is in accordance with the observed impact of this training on the functional properties of the three types of MUs. Namely, significant changes in contractile parameters were found predominantly in FR MUs (Fig 3 and Table 1). This indicates that the applied training involved mainly the S and FR MUs, which are present in a higher proportion in the MGS than in the MGF [40–42].

Interestingly, a progressive increase in the percentage of FR MUs, and a concomitant decrease in FF MUs through the training program (Fig 2), indicates a physiological transformation of FF into FR MUs, leading to the increased fatigue resistance of the muscle. This suggests that neuromuscular adaptive changes that occur in the early stage of training may lead to enhanced muscle efficiency and attenuation of metabolic disturbances during exercise. This adaptive response of muscle is consistent with some earlier observations showing that training-induced enhancement of muscle metabolic stability during exercise precedes muscle mitochondrial biogenesis in humans [8,12,19]. An increase in muscle efficiency at this stage of training, i.e., before mitochondrial biogenesis, may constitute an adaption of the system to decreased muscle fatigue and enhanced muscle performance (for review see [4]).

In the current study, we aimed to answer the question whether the observed functional changes in MUs result from training-induced enhancement of muscle energy status, or whether they represent adaptive responses induced by the shortage of muscular energy during subsequent bouts of endurance training. Due to the pattern of changes observed in mitochondrial biogenesis markers such as an increase in mitochondrial ETC protein content, the second option appears to be more likely, i.e., training-induced adaptive changes lead to energy saving and enhanced muscle efficiency in the early stages of training before intensification of mitochondrial biogenesis. However, we cannot exclude a possibility that the adaptive neuromuscular changes could result from training-induced enhancement of muscle energy status during exercise, with no involvement of mitochondrial biogenesis-related mechanisms, such as intensification of parallel activation [5,8,12,19]–known also as each step-activation (ESA) [69], and/or via an increase in mitochondrial efficiency (an increase in P/O ratio) [15], allowing the observed improvement in MU function (Fig 3B). This rather complex adaptive changes observed in neuromuscular function, including increased fatigue resistance, shortening of the twitch contraction time and increased ability to potentiate force in the early stages of training, is a new observation which requires further study.

## Conclusions

The present study showed for the first time that as little as 2 weeks of endurance training is sufficient to induce pronounced changes in the contractile properties of MUs predominantly of the FR type. These early muscle functional changes in MU properties are accompanied by activation of signalling within mitochondrial network reorganisation, including an increase in expression of *Mfn1*, *Opa1* and increase in PGC-1α in the MGS. Interestingly in the early stage of training this muscle adaptive response is present in an absence of changes in the markers of mitochondrial biogenesis such as mitochondrial DNA copy number (mtDNA/nDNA) and the amount of the electron transport chain components—which in our study were clearly visible only in the latter stage of training. Therefore, we conclude that enhancement of MU functional properties is a rapid adaptive response, which precedes an increase in mitochondrial ETC protein content, but is associated with an activation of signalling that controls the muscle mitochondrial network reorganisation and mitochondrial biogenesis.

## Supporting information

S1 Table Training schedule. (DOCX)

S1 File All data for Table 1 and values of the 20 Hz index. (XLSX)

S2 File Body mass and medial gastrocnemius muscle mass. (XLSX)

S3 File All data for Fig 2. (XLSX)

S4 File Changes in tetanus force during the fatigue test. (XLSX)

S5 File All data for Fig 3. (XLSX)

S6 File All data for Fig 4. (XLSX)

S7 File All data for Table 2. (XLSX)

S8 File All data for Table 3. (XLSX)

S9 File All data for Fig 5. (XLSX)

S10 File All data for Fig 6. (XLSX)

S11 File All data for Fig 7. (XLSX)

S12 File All data for Fig 8A. (XLSX)

S13 File All data for Fig 8B. (XLSX)

S14 File All data for Fig 9. (XLSX)

S15 File NC3Rs ARRIVE guidelines checklist. (PDF)
