# Supplementary material for: Adaptation of motor unit contractile properties in rat medial gastrocnemius to treadmill endurance training: Relationship to muscle mitochondrial biogenesis
Source: PLoS One. 2018 Apr 19;13(4):e0195704. doi: 10.1371/journal.pone.0195704 (PMC5908179; doi:10.1371/journal.pone.0195704)
Supplement: S1 Table — (DOCX) [file pone.0195704.s001.docx]

| First week  (conditioning) | 1) 5-10 min slow running at 5-10 m/min with 5-10 s episodes of progressive increase of treadmill speed up to 30-40 m/min followed by decrease of the velocity, repeated 10-20 times;  2) 60 min rest;  3) repeated point 1) of procedure |
| --- | --- |
| Second week | 40 min continuous run at velocity (v) = 30 m/min |
| Third week | 60 min continuous run at v = 30 m/min |
| Fourth week | 60 min continuous run at v = 30 m/min, with the acceleration every 10 min to v = 35 m/min for 30 s (6 accelerations in 10, 20, 30, 40, 50, 60 minute) |
| Fifth week | 60 min continuous run at v = 30 m/min, with the acceleration every 10 min to v = 40 m/min for 30 s (6 accelerations in 10, 20, 30, 40, 50, 60 minute) |
| Sixth week | 60 min continuous run at v = 30 m/min, with the acceleration every 10 min to v = 40 m/min for 30 s (6 accelerations in 10, 20, 30, 40, 50, 60 minute) |
| Seventh week | 80 min continuous run at v = 30 m/min, with the acceleration every 13 min to v = 40 m/min for 30 s (6 accelerations in 13, 26, 39, 52, 65,78 minute) |
| Eight week | 80 min continuous run at v = 30 m/min, with the acceleration every 13 min to v = 40 m/min for 30 s (6 accelerations in 13, 26, 39, 52, 65, 78 minute) |
